# Persistent symptoms, cognitive impairment, and clinical predictors of long COVID one year after Omicron infection: A clinical case–control study from the Faroe Islands

**DOI:** 10.1371/journal.pone.0351564

**Published:** 2026-06-16

**Authors:** Gunnhild Helmsdal, Marnar Fríðheim Kristiansen, Eyðbjørg Klemmentsen Gaard, Barbara Joensen Eysturoy, Pál Weihe, Eina Hansen Eliasen, Maria Skaalum Petersen

**Affiliations:** 1 Department of Research, Tórshavn, Faroe Islands; 2 Medical Department, National Hospital, Tórshavn,‌‌ Faroe Islands; 3 University of the Faroe Islands, Tórshavn,‌‌ Faroe Islands; Médecins Sans Frontières (MSF), SOUTH AFRICA

## Abstract

**Background:**

Six years since the emergence of SARS-CoV-2, the newer variants of the virus continue to have long-term health effects.

**Objectives:**

The aim of the study was to investigate persistent symptoms, cognitive impairment, and clinical and paraclinical predictors of long COVID in individuals infected during the Omicron wave.

**Methods:**

We conducted a clinical case-control study including participants with persistent symptoms up to 13 months after confirmed SARS-CoV-2 Omicron infection (long COVID or LC group) and antibody-verified never-infected controls (NI group).

**Results:**

A total symptom score based on a 24-item questionnaire was strongly associated with increased odds of long COVID (adjusted odds ratio (aOR) 1.21, 95% CI 1.13–1.30, p < 0.001). Sub-analysis showed particularly strong associations for fatigue, cognitive impairment, neurological symptoms, and symptoms from the cardiopulmonary and musculoskeletal systems. Both mental impairment and fatigue independently predicted long COVID (aOR 1.27, 95% CI 1.14–1.42, p < 0.001, and aOR 1.27, 95% CI 1.11–1.46, p < 0.001, respectively). Additionally, a higher number of self-reported infections during the follow-up period increased the odds of long COVID (aOR 1.57, 95% CI 1.06–2.34, p = 0.025), though this was not reflected in antibiotic use. Finally, blood analyzes showed that lower white blood cell counts were associated with increased odds of long COVID in women, but not in men, however the clinical significance of this finding remains uncertain.

**Conclusions:**

One year after Omicron infection, a subset of people continue to experience a substantial symptom burden, particularly fatigue, cognitive impairment, and mental well-being, and a higher frequency of intercurrent infections.

## Introduction

It has been nearly six years since the beginning of the pandemic, during which an estimated 7.1 million deaths have been attributed to COVID-19 [1], contributing to a decrease in life expectancy across 204 countries as well as causing long-term debilitating post-infectious symptoms [2]. The Omicron variant was first detected in November 2021 and rapidly gained dominance to become the cause of most COVID-19 cases worldwide and in the Faroe Islands. While the Omicron variant has a milder course of disease in most cases, knowledge about potential long COVID after infection with the Omicron variant is limited.

Post-acute sequelae of SARS-CoV-2, post-COVID-19 condition, or long COVID are conditions of long-term symptoms, which studies show are experienced by approximately 10% of people after infection with SARS-CoV-2, albeit depending on the variant of concern [3,4]. The latest definition by the National Academies of Sciences, Engineering, and Medicine (NASEM) states that long COVID is an infection-associated chronic condition that occurs after SARS-CoV-2 infection and is present for at least 3 months as a continuous, relapsing, and remitting, or progressive disease state that affects one or more organ systems [5]. Global estimates suggest that approximately 6 in 100 people with COVID-19 develop long COVID, although estimates vary widely, particularly in early pandemic cohorts [6]. The etiology of long COVID is still somewhat unclear, but many theories have been presented, including viral persistence, reactivation of latent viruses such as Epstein-Barr virus, low-grade inflammation, autoimmunity, and dysregulation or dysfunction of the immune response, the endothelium, the coagulation system, endocrine functions, mitochondria, and more [7].

An increased risk of developing long COVID has been associated with old age, female sex, medical comorbidities, and a severe course of acute disease [7–9]. Also, the ancestral and the following variants of SARS-CoV-2 have shown a higher incidence of long COVID compared to the later, more transmissible types of Omicron variants [10,11]. Reinfection with the virus is suspected to increase the risk of long COVID, but further studies are needed [12]. A protective effect against long COVID has been observed with vaccination prior to infection, but it does not seem to influence ongoing prolonged symptoms [13]. Also, treatment with Nirmatrelvir in certain risk groups seems to reduce the risk of long COVID [14].

Studies are emerging assessing the risk and outcome of long COVID following infection with the Omicron variant, but only a few of these studies have included a control group with uninfected people, mainly because very few people are “COVID-19-naïve” today. Including a control group is important, as it can prevent misclassification of symptoms due to factors other than a COVID infection as long COVID.

The Faroe Islands are an isolated archipelago in the North Atlantic consisting of 18 islands with approximately 55,000 inhabitants. The islands have a modern health care system and an integrated health registry enabling excellent epidemiological and clinical studies regarding COVID-19 [15–19]. Due to the isolated setting and comparatively limited spread of COVID-19 in 2020 and 2021, as well as a high amount of testing, it was uniquely possible to identify a group of non-infected individuals, which could be included as a control group.

Here, we present a clinical follow-up study of people with long COVID symptoms following infection in January 2022, compared with a control group not previously infected with SARS-CoV-2. In addition to self-assessed symptom burden and mental impairment, we aimed to assess whether other predictors were associated with long COVID by including a general and neurological assessment of the participants. We also included paraclinical tests such as heart and lung function, and blood samples to assess whether these markers of organ and immune system functions could predict long COVID. Finally, we also investigated whether infections and antibiotic use following COVID-19 could predict long COVID.

## Materials and methods

### Population and recruitment

A large proportion (n = 14,666) of the population of the Faroe Islands was infected with RT-PCR-confirmed COVID-19 in January 2022 (n = 53,560 as of January 31, 2022). They all received an online invitation in February 2022 to answer a questionnaire. A control group, individuals who had not tested positive for SARS-CoV-2 in December 2021 and January 2022 and had a previous negative serology test in two former seroprevalence studies conducted in April 2020 [18] and November 2020 [19], were also invited. Recruitment has been described previously, and 4506 infected people and 530 controls answered the first questionnaire [17]. During the next six months, three subsequent questionnaires were sent.

Based on the last questionnaire, 6 months after enrolling in the study (in July 2022), all cases who either reported having symptoms affecting their daily life in a moderate or severe sense or answered having either partly or not recovered, were invited to a clinical follow-up study (long COVID or LC group). Controls who had answered that they had not had SARS-CoV-2 infection at the last follow-up were also invited to a clinical examination (never-infected or NI group).

All participants were first contacted by e-mail with an invitation to participate in the clinical study. If they agreed, an appointment was made by phone for a clinical follow-up that was conducted in November 2022 to February 2023.

### Questionnaires

The first questionnaire (baseline) included questions regarding symptoms during the disease period and vaccination status, as well as background questions about general health, morbidities, medicine intake, education and occupation, and weight and height. The subsequent questionnaires included questions about lasting symptoms after the infection had passed, to assess the prevalence of long COVID, as well as questions about self-evaluated recovery and the frequency of respiratory infections since the infection with SARS-CoV-2.

During a personal interview at the clinical follow-up, all participants (long COVID and never-infected group) answered a 24-item questionnaire about current symptoms, a 9-item questionnaire regarding mental impairment, and an 8-item fatigue impairment questionnaire (Daily Fatigue Impact Scale [20]). In all three questionnaires, the symptoms were rated from 0 to 3 (none, mild, moderate, or severe); a high score indicates severe symptoms or impairment. The participants were also asked about the number of respiratory infections with fever during the previous year and about self-evaluated recovery following the SARS-CoV-2 infection (fully, partly, or not recovered) (LC group only).

The questionnaires are shown in S1-S3 Tables in S1 File. The 24-item symptom questionnaire was developed based on the knowledge of long COVID. The 9-item mental impairment questionnaire was developed in collaboration with a certified clinical psychologist to assess neurocognitive and emotional aspects, including items on memory and concentration before and after COVID-19, as well as mood, stress, and anxiety. The 8-item fatigue impairment questionnaire is a validated tool assessing fatigue [20].

### Clinical and paraclinical examinations

The clinical examination included measurement of weight, height, blood pressure, and auscultation of the heart and lungs. The neurological assessment covered cranial nerves, general muscle strength, including hand grip strength using a manual dynamometer, general sensitivity, and reflexes. Peripheral tactile sensitivity was evaluated by using monofilaments on the plantar side of the 1^st^ toe of both feet (0.004–300 g), and vibration sense with a biothesiometer on the 1^st^ toe of both feet (vibration perception threshold in volts). Smell was tested using the 16-item Sniffin’ Sticks panel (Burghart Messtechnik, Germany) where participants had four optional answers to each smell, taste with four Taste Strips (salty, sweet, sour, bitter; Burghart Messtechnik, Germany). A cognitive test was also performed (The Montreal cognitive assessment, MoCA) [21].

The paraclinical examination included a 12-lead electrocardiogram (ECG) with a portable ECG machine (Edan) with the ECGs classified according to the Minnesota system (normal, minor abnormality, major abnormality). A lung function test (Medikro spirometer) was also performed with spirometry values including forced expiratory volume in the first second (FEV1), forced vital capacity (FVC), and FEV1/FVC, expressed as percentages of predicted values by sex, age, and height. Blood samples were analyzed at the Laboratory of the National Hospital of the Faroe Islands for red and white cell count, kidney and liver function, electrolytes, ferritin, C-reactive protein, cholesterol, vitamin B12, vitamin D, HbA1c, TSH, T3, T4, and cortisol.

A quantitative immunoassay of antibody status was performed to assess whether the controls, all vaccinated, could have had an asymptomatic SARS-CoV-2 infection, measuring antibodies against the N protein and the RBD protein [22]. Levels above a defined threshold indicated previous infection. The immunoassay has both a high sensitivity and a high specific, hence very few controls are expected to be either false-positive or false-negative [22]. Information regarding prescribed antibiotic use (local, oral, and intravenous) during the previous 12 months was obtained through the integrated patient journal system.

### Ethics declaration

The study was approved by the Faroese Research Ethics Committee and performed according to the Data Protection Act. Written informed consent to participate was obtained from all participants.

### Statistical analysis

Descriptive results were presented with median and range for continuous variables and with numbers and percentages for categorical variables. We used the independent-samples t-test and the chi-square test to compare the groups. Multivariate logistic regression was used to evaluate symptom scores, clinical data, blood markers, self-reported number of infections, and antibiotic use associated with odds of long COVID, taking these potential confounders into account: sex (female/male), age (continuous), smoking (ever/never), body mass index (BMI, continuous), self-reported daily medication use (yes/no) and chronic diseases (yes/no). The potential confounder variables included in the analysis were considered based on previous knowledge of COVID-19 epidemiology and potential confounders [23]. Potential confounders were included in a multivariable regression model and subjected to backward selection; variables with p-values < 0.05 were retained in the final model. The analysis showed an association between age, sex, comorbidities, and medication with the analyzed variables, and were included in the multivariable logistic regression analysis. Results from the multivariable logistic regression models were presented as adjusted odds ratios (aORs), respectively, together with 95% confidence intervals (CIs)

Due to a difference in the reference intervals for men and women, the analyses of blood sample results were done stratified by sex.

Long COVID was defined as a condition occurring after SARS-CoV-2 infection, with symptoms present at least 3 months after infection.

Assuming a two-sided α of 0.05 and a small-to-moderate effect size, a sample size of 200 participants per group provided 80% power to detect a clinically meaningful difference from the examinations.

For the statistical analyses, participants from the case group reported having recovered, and participants from the control group who had antibodies indicating previous infection were excluded. Sensitivity analyses were performed to assess differences between included and excluded participants and whether this affected the multivariable analysis of long COVID.

No correction for multiple comparisons was applied as the analyses were considered exploratory.

All statistical analyzes were performed using SPSS version 25. P (two-tailed) < 0.05 was considered statistically significant.

## Results

Of the 293 eligible people previously infected, 200 accepted to participate, 92 were either unreachable or did not wish to participate, and one was excluded due to a language barrier, resulting in a 68% participation rate. At follow-up, 23 reported having fully recovered; the final LC study group consisted of 177 individuals.

Of the 112 eligible controls, 93 participated, 19 were either unreachable or did not wish to participate, resulting in an 83% participation rate. Of the controls, 28 (30.1%) had antibody levels against the N protein corresponding to previous infection and were excluded from the analysis. Hence, the final NI group consisted of 65 individuals.

**Fig 1:**
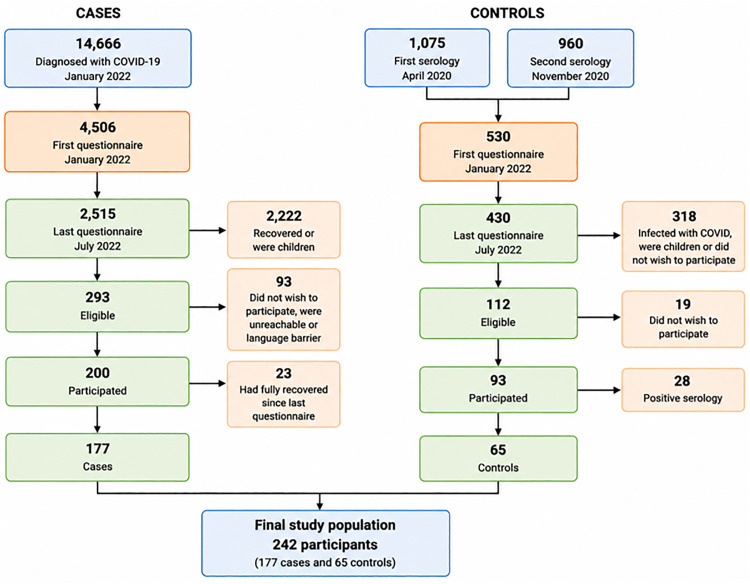
Flow chart showing the recruitment of cases and controls.

Fig 1 shows the cases and controls in a flow chart presenting the recruitment process.

Clinical characteristics are presented in Table 1. The groups differed statistically in terms of sex, age, daily medication use, and vaccination status. The long COVID group reported significantly more symptoms across the three questionnaires, resulting in higher scores in all three modalities. The long COVID group had a mean follow-up of 11 months (range 9.5 to 13 months). Clinical characteristics of men and women separately are shown in S4 Table in S1 File.

**Table 1:** Characteristics of the long COVID group and the never-infected group in the Omicron long COVID study.

	Long COVID group (n = 177)	Never-infected group (n = 65)	P-value^*^
Sex, women, n (%)	134 (75.7)	27 (41.5)	<0.001
Age, years, mean (SD)	44.6 (13.7)	60.6 (12.0)	<0.001
Ever smoker, n (%)^1^	96 (54.9)	42 (64.6)	0.2
Body mass index (kg/m^2^), mean (SD)	29.4 (5.9)	29.7 (5.1)	0.8
Self-reported daily medication use, n (%)^2^	71 (40.8)	39 (60.9)	0.006
Self-reported chronic disease, n (%)^3^	88 (49.7)	41 (63.1)	0.07
Hospitalization, n (%)	0	NA	NA
Follow-up time, mean (SD)	10.9 (0.9)	NA	NA
COVID-19 vaccination, n (%)			0.04
0	11 (6.2)	0	
2 or 3	166 (93.8)	65 (100.0)	
Total symptom score, mean (SD; range)	20.0 (10.1; 2-57)	7.2 (6.5, 0-26)	<0.001
Total fatigue score, mean (SD; range)	10.0 (6.0; 0-24)	2.8 (4.9; 0-21)	<0.001
Total mental impairment score, mean (SD; range)	7.4 (5.6; 0-27)	2.6 (3.8; 0-19)	<0.001
Number of self-reported infections, mean (SD)	1.8 (1.1)	0.7 (0.7)	<0.001
Number of treated infections, mean (SD)	0.9 (1.4)	0.8 (1.3)	0.4

Abbreviation: SD: Standard deviation

* Pearson Chi-square for binary variables, t-test for continuous variables, and multivariable logistic regression analysis for scores and infectious episodes (adjusted for age, sex, comorbidities, and medication) ** for the long COVID group, before SARS-CoV-2 infection, for the never-infected group, one year prior to the examination

^1^two missing in long COVID group

^2^three missing in the long COVID group, one missing in the never-infected group

^3^asthma, heart disease, carnitine transporter deficiency, inflammatory bowel disease, hypertension, hypercholesterolemia, chronic obstructive pulmonary disease, type 2 diabetes

The most common symptoms reported by the long COVID group were fatigue (97%), headache (77%), problems with sleep (74%), problems with concentration (72%), problems with memory (69%), and symptoms from the respiratory system (62%), whereas the most common symptoms reported by the never-infected group were symptoms from the respiratory system (39%), arthralgia (38%), fatigue (35%), and problems with sleep (32%).

**Fig 2:**
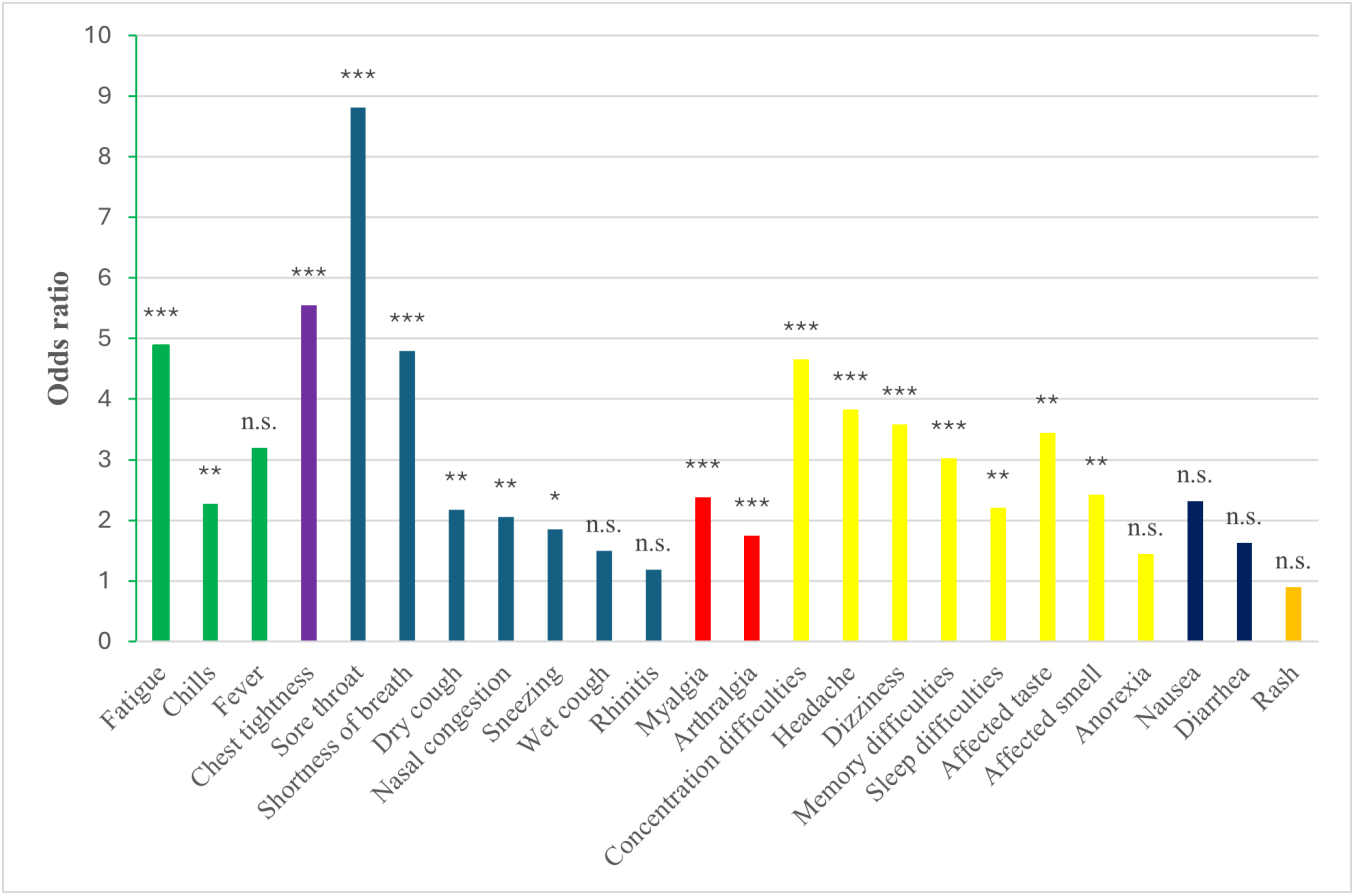
Odds ratio for 24 different symptoms in the LC group compared to the NI group. The colors indicate the group of symptoms: green constitutional, purple cardiac, blue pulmonary, red musculoskeletal, yellow neurological, black gastrointestinal, and orange dermatological. The analyses were adjusted for age, sex, comorbidities, and medication. *** p < 0.001, ** p < 0.01, * p < 0.05, n.s not significant.

Total symptom score was associated with increased odds of long COVID (aOR 1.21, 95% CI 1.13–1.30, p < 0.001). Sub-analysis indicated stronger associations in fatigue, cognitive impairment, and other neurological symptoms, but also with symptoms from the cardiopulmonary and the musculoskeletal systems. Fig 2 shows the distribution of the different symptoms with odds ratios for long COVID for each symptom separately. Detailed results are shown in S5 Table in S1 File.

In the mental impairment questionnaire, the long COVID group notably reported difficulties with concentration (73%) and memory (72%), feeling more stressed (71%), experiencing post-exertional fatigue (67%), and being somewhat less affected in emotions, depression, and anxiety. In contrast, the never-infected group showed a more even distribution across these areas, although they were mostly affected in memory (27%), feeling more stressed (26%), or depressed (25%). S1 Fig in S1 File shows the distribution of the reported symptoms. Mental impairment score was associated with increased odds of long COVID (aOR 1.27, 95% CI 1.14–1.42, p < 0.001). This was especially seen in problems with memory and concentration, and feeling exhausted after exertion or feeling stressed, less with feeling emotional, depressed, or anxious (Table 2).

**Table 2:** Associations between long COVID and the total score or the scores from the individual questions in the mental impairment symptoms questionnaire.

	aOR	95% CI	P-value*
*Total score*	*1.27*	*1.14-1.42*	*0.001*
Memory difficulties	3.30	1.85-5.90	<0.001
Previous memory difficulties**	1.47	0.71-3.04	0.3
Concentration difficulties	5.40	2.71-10.77	<0.001
Previous concentration difficulties**	1.78	0.78-4.07	0.2
Post-exertional fatigue	3.92	2.28-6.75	<0.001
More emotional	2.10	1.26-3.52	<0.01
More depressed	1.77	1.10-2.85	<0.05
More stressed	2.40	1.49-3.85	<0.001
More anxious	1.93	1.05-3.54	<0.05

Abbreviations: OR: Adjusted Odds ratio; CI: Confidence Interval

* Multivariable logistic regression analysis, adjusted for age, sex, comorbidities, and medication ** for the LC group, before SARS-CoV-2 infection, for the NI group, one year prior to the examination

In the fatigue impact questionnaire, which was only answered by the participants who reported fatigue in the symptom questionnaire, (n = 171 from the long COVID group and n = 23 from the never-infected group), the most common problems reported in both groups were less motivation, physical effort, and physical activity, but also problems with alertness in the long COVID group (S2 Fig in S1 File). The total fatigue impairment score (from the Daily Fatigue Impact Score) was, as expected, significantly associated with increased odds of long COVID (aOR 1.27, 95% CI 1.11–1.46, p < 0.001). However, the sub-analyses with the individual questions did not show a similar statistically significant association, except for less alertness and a trend for fatigue due to physical effort (Table 3).

**Table 3:** Associations between long COVID and the total score or the scores from the individual questions in the Daily Fatigue Impact questionnaire (D-FIS)^124^.

	aOR	95% CI	P-value*
*Total score*	*1.26*	*1.15-1.38*	*0.001*
Less alert	2.25	1.19-4.25	<0.05
Reduced workload	1.59	0.94-2.70	0.08
Less motivated	1.62	0.95-2.76	0.08
Physical effort	1.74	0.99-3.07	0.06
Making decisions	1.13	0.65-1.96	0.7
Finishing tasks	1.71	0.92-3.18	0.09
Slow thinking	1.44	0.81-2.58	0.2
Physical activities	1.54	0.82-2.89	0.2

Abbreviations: aOR: Adjusted Odd ratio; CI: Confidence Interval

* Multivariable logistic regression analysis adjusted for age, sex, comorbidities, and medication

Of note, a sex difference was seen regarding the above-mentioned scores. The women in the long COVID group reported higher total scores than men in all of these three questionnaires, hence higher odds of long COVID were seen with female sex (aOR 1.06, 95% CI 1.01–1.11, p = 0.01 for total symptom score, aOR 1.08, 95% CI 1.00–1.17, p = 0.046 for mental impairment score, and aOR 1.12, 95% CI 1.04–1.20, p = 0.002 for fatigue impairment score). This association was not observed in the NI group with any of these scores.

In the questionnaire, the participants also reported the number of self-evaluated respiratory infectious episodes through the preceding year. The long COVID group had a mean number of self-reported infections of 1.8, compared with 0.7 in the never-infected group (Table 1). The number of self-reported infections was associated with increased odds of long COVID (aOR 1.57, 95% CI 1.06–2.34, p = 0.025) – meaning 57% increased odds of long COVID per additional infectious episode. The women in the long COVID group reported more infections than the men (p < 0.001), with no statistical difference seen in the never-infected control group (p = 0.1).

To evaluate whether this could be reflected in the use of antibiotics in the same period, the number of prescribed antibiotic treatments was found in the electronic health journal system. When looking at total antibiotic use (local, oral, and intravenous treatments), only a trend of an increased number of treatments was seen associated with long COVID (aOR 1.71, 95% CI 0.81–3.61, p = 0.159). When looking at the antibiotic treatments in groups of infections, e.g., upper, or lower respiratory infections, upper or lower urinary infections, gastrointestinal, local infections, etc., no associations were seen, but the most prevalent infections were in the respiratory system (results not shown). However, again, a sex difference was seen, as the women in the LC group had received significantly more antibiotic treatments than the men (p < 0.01), with no statistical difference seen in the NI group (p = 0.2).

Analyzing the blood results separately for men and women (due to possible differences in reference intervals and other sex-dependent factors), the biochemical blood results showed a difference in the white blood cell count, where the LC women had lower counts of total and differentiated leukocytes compared to the NI women, e.g., mean values of 6.7 versus 7.2 x 10^9^ per liter in total number of leukocytes, respectively (S7 Table in S1 File). No difference was seen in men (S6 Table in S1 File). Hence, a higher white blood cell count was associated with decreased odds of long COVID in women (aOR 0.66, 95% CI 0.49–0.89, p = 0.006), or, alternatively, using an inverted OR, a one-unit decrease in leukocytes was associated with 1.52-fold higher odds of long COVID in women. Similar results were found within the differential count, where all cells were lowered in the long COVID group, hence higher neutrophils, basophils and lymphocytes were significantly associated with lower odds of long COVID (aOR 0.66, 95% CI 0.45–0.97, p = 0.036, aOR 0.000001, 95% CI 0.00–0.08, p = 0.016, and aOR 0.41, CI 0.19–0.88, p = 0.023, respectively). No association was seen with eosinophils or monocytes. Although the values were lower compared to the NI group, very few LC women had any clinically defined mild leukopenia, neutropenia, or lymphopenia and the NI group did not have any outliers either. These differences were not seen with the men. S6 and S7 Tables in S1 File show white blood cell counts for men and women in the LC and NI groups, respectively.

No differences were seen in other blood analyses, including acute phase reactants such as thrombocytes, ferritin, or C-reactive protein (results not shown). The participants also underwent a thorough clinical and paraclinical evaluation as described in the methods section, but no associations were seen with long COVID.

### Sensitivity analysis

To assess whether excluding recovered and seropositive participants could be justified, we conducted a sensitivity analysis. In the case group, we found that the recovered participants were significantly older and had less daily medication use. They scored lower on all three questionnaires and reported fewer infections than the non-recovered participants. In the control group, we found that the seropositive participants were significantly younger but were otherwise similar in baseline characteristics. We found no difference in questionnaire scores, but the group reported more infections in the previous year than the seronegative group. These observations supported our choice of outcome‌‌ groups.

## Discussion

Although the individual risk of long COVID has decreased in recent years, likely due to vaccination, prior immunity, and changes in circulating variants, the global prevalence of long COVID is still high [6,12–14]. As shown in multiple international studies, infection with the Omicron variant also carries a risk of long COVID [10,24–27], as our findings confirm.

Compared to a COVID-naïve control group, participants continued to report significantly higher symptom burden up to 13 months post-infection, particularly with fatigue, headache, problems with sleep, respiratory symptoms, as well as cognitive impairment, and reduced mental well-being. This was evident despite the cases being younger and healthier than the controls. The types of symptoms also differed between the groups, with controls reporting mostly respiratory symptoms, probably mostly reflecting the winter season, and some chronic symptoms such as fatigue and arthralgia, but with a much lower prevalence than the cases. We also found that both the mental impairment and the fatigue impairment questionnaires reflected problems with cognition and fatigue in the long COVID group rather than emotional, depressive, or anxiety symptoms that were more equally distributed in the control group. These results are consistent with those of other case-control studies, including those on the Omicron variant [28–30]. The study by Asakura et al. was a cross-sectional case-control study in Sapporo, Japan, which assessed symptoms following infection with different strains of SARS-CoV-2 at five time points (n = 8,018). At 13−18 months post-infection, the most prevalent symptoms were systemic, respiratory, and neuropsychiatric [28]. The study by Iba et al. in the metropolitan area of Tokyo, Japan, included cases and controls from the Omicron-dominated period (n = 14,710) with a mean follow-up time of approximately 6 months. The cases in this study had predominantly had a mild SARS-CoV-2 infection, and the most common persisting symptoms compared to the control group were neurological (smell and taste), systemic (fatigue), and cognitive impairment [29]. A large case-control study from Shanghai, China, including 21,799 individuals from the Omicron B.A.2 period, assessed the participants at 6 and 12 months post-infection, showing a higher likelihood of cough and fatigue with the cases compared to the controls, but the study was limited by a low number of controls (n = 979) [30]. These case-control studies of persisting symptoms after infection with the Omicron variant are consistent with studies of other variants, as presented in a comprehensive systematic review by Franco et al., which included 63 controlled cohort studies [31]. The review showed that, regarding persistent symptoms, individuals with documented SARS-CoV-2 infection had an increased risk of having two or more persistent symptoms at follow-up, especially those related to neurological clusters [31].

Our study also showed that self-reported infectious episodes in one year following the infection with SARS-CoV-2 were a predictor of long COVID, with 57% increased odds of long COVID per infectious episode, suggesting increased susceptibility to infections. However, this was not reflected in the antibiotic use in the same period, indicating that the infections could have been self-limiting, such as upper respiratory viruses or mild bacterial infections not needing treatment. This may reflect the broader post-pandemic surge in respiratory pathogens that was seen in the winter seasons following the lifting of restrictions, especially in non-immune small children [32]. This has been named “immunity debt”, as the immune system has had less exposure to pathogens during lockdown and subsequent restrictions in society. Another hypothesis, “immunity theft”, proposes that COVID-19 might impair the immune response and increase susceptibility to later disease [33]. Evidence supporting this remains preliminary, though large cohort studies have observed higher risks of secondary infections and hospitalization following COVID-19. One example is a large, matched cohort study by Hou et al from the UK Biobank (5,151 cases and 51,402 controls) where COVID-19 patients had increased subsequent risks of life-threatening secondary infections in a 3-month period – to an equal extent or beyond risk elevations observed for patients with seasonal influenza [34]. Another large cohort study by Xiang et al. (n = 412,096) revealed an increased risk of hospitalization for a wide range of pulmonary and extra-pulmonary diseases following COVID-19, especially for severe infections [35]. Finally, the association between long COVID and a higher number of self-reported infections could be explained in part by recall bias, whereby individuals with long COVID may over-report past infectious episodes.

In women, total and differential white blood cell (WBC) counts differed between those with long COVID and the never-infected controls, and WBC measures were significantly associated with increased odds of long COVID in multivariable logistic regression. Several studies have reported persistent alterations in immune function after COVID-19, including changes in complement and coagulation pathways and in specific T-cell subsets, which could reflect a chronic inflammatory state and partly explain long-term symptoms [36–40]. Severe neutropenia and lymphopenia are also well described during acute severe COVID-19 but typically resolve after recovery [41]. Case-control studies examining WBC counts in post-COVID cohorts have produced divergent results, and these findings have not yet been integrated into a unified mechanism for long COVID [42,43]. Although we observed a statistically significant association in women, we did not find that mean WBC values in long COVID women and men were substantially different; rather, the control women tended to have higher mean values than control men, suggesting that baseline differences between groups (e.g., chronic disease) could contribute to the observed association and therefore be a result of residual confounding. The extremely low odds ratio and a corresponding very broad confidence interval for the association between basophils and long COVID are indicative of model instability and further caution towards these results.

Sex-specific patterns in COVID-19 are well-known in the literature: men are more prone to severe acute disease and higher mortality, whereas women appear more susceptible to developing long COVID and report a higher symptom burden [44–47]. Consistent with this, our data show a higher prevalence of long COVID among women, a higher symptom burden (general symptoms, mental impairment, and fatigue), and more post-COVID infections.

Although the participants with long COVID reported significantly more general symptoms and also had significantly higher scores for mental and fatigue impairment compared to the group of never-infected individuals, a difference in objective measures in the clinical evaluation could not be found.

In the systematic reviews by Salman et al. (n = 3,041) and Rahmati et al. (1,436 cases and 958 controls), cardiorespiratory and cardiac functions were decreased in patients with long COVID [48,49]. Also, in a meta-analysis by Panagea et al., including 36 studies, persistent neurocognitive impairment was detected in individuals with long COVID [50]. However, the majority of these studies included individuals who had been hospitalized and who had been infected in the pre-Omicron period; therefore, it may be difficult to assess and identify objective measures of long COVID in people who have mainly had mild or moderate acute disease.

The study benefits from a well-defined nationwide cohort, seronegative controls, and a comprehensive clinical assessment. However, the limited size of the control group, especially after excluding seropositive individuals, limits the statistical power of the comparisons. Also, the lack of pre-infection baseline data limits inference and generalizability. Furthermore, the case-control design could be susceptible to residual confounding and recall bias, with difficulty establishing a cause-and-effect relationship.

## Conclusions

One year after Omicron infection, a subset of individuals continues to have a substantial symptom burden, particularly fatigue, cognitive and mental impairment, and a higher frequency of infections. Also, women seem to have a higher risk of and be more affected by long COVID than men. Studies are ongoing, and in the following years, a clearer view of the long-term effects of SARS-CoV-2 will hopefully emerge.

## Supporting information

S1 File Supporting Tables. (DOCX)
