# Supplementary material for: Persistent symptoms, cognitive impairment, and clinical predictors of long COVID one year after Omicron infection: A clinical case–control study from the Faroe Islands
Source: PLoS One. 2026 Jun 16;21(6):e0351564. doi: 10.1371/journal.pone.0351564 (PMC13271491; doi:10.1371/journal.pone.0351564)
Supplement: S1 File — (DOCX) [file pone.0351564.s001.docx]

**S1 Table** Symptom questionnaire

| Have you experienced any of these symptoms during the previous week? | None | Mild | Moderate | Severe |
| --- | --- | --- | --- | --- |
| 1.1 Dry cough |  |  |  |  |
| 1.2 Wet cough |  |  |  |  |
| 1.3 Sneezing |  |  |  |  |
| 1.4 Fever |  |  |  |  |
| 1.5 Headache |  |  |  |  |
| 1.6 Sore throat |  |  |  |  |
| 1.7 Shortness of breath |  | 1 |  |  |
| 1.8 Chest tightness |  |  |  |  |
| 1.9 Congested nose |  |  |  |  |
| 1.10 Rhinitis |  |  |  |  |
| 1.11 Muscle ache |  |  |  |  |
| 1.12 Joint ache |  |  |  |  |
| 1.13 Chills |  |  |  |  |
| 1.14 Affected smell |  |  |  |  |
| 1.15 Affected taste |  |  |  |  |
| 1.16 Affected appetite |  |  |  |  |
| 1.17 Diarrhea |  |  |  |  |
| 1.18 Nausea |  |  |  |  |
| 1.19 Fatigue |  |  |  |  |
| 1.20 Rash |  |  |  |  |
| 1.21 Dizziness |  |  |  |  |
| 1.22 Affected memory |  |  |  |  |
| 1.23 Affected concentration |  |  |  |  |
| 1.24 Affected sleep |  |  |  |  |

2.1 If you are still having symptoms, are they affecting your daily activities?

No Yes, some Yes, a lot Don’t know

2.2 Do you feel that you have recovered after having COVID-19?

No, not at all No, not completely Yes, completely

**S2** **Table** Mental health questionnaire

| Mental health questions | None | Mild | Moderate | Severe |
| --- | --- | --- | --- | --- |
| 3.1 Today, do you have problems with your memory? |  |  |  |  |
| 3.2 Before COVID-19, did you have problems with your memory? |  |  |  |  |
| 3.3 Today, do you have problems concentrating? |  |  |  |  |
| 3.4 Before COVID-19, did you have problems concentrating? |  |  |  |  |
| 3.5 Have you been feeling extremely unwell/tired after physical or mental exertion? |  |  |  |  |
| 3.6 Have you been feeling more sensitive post-COVID/since January 2022? |  |  |  |  |
| 3.7 Have you been in a bad/worse mood post COVID/since January 2022? |  |  |  |  |
| 3.8 Have you been feeling more stressed post-COVID/since January 2022? |  |  |  |  |
| 3.9 Have you been feeling more anxious post-COVID/since January 2022? |  |  |  |  |

**S3 Table** Fatigue Impact Scale

| Fatigue (if yes to question 1.19) | No problem | Small problem | Moderate problem | | Great problem |
| --- | --- | --- | --- | --- | --- |
| 4.1 Due to fatigue, I feel less alert |  |  |  |  | |
| 4.2 Due to fatigue, I must reduce my workload or responsibilities |  |  |  |  | |
| 4.3 Due to fatigue, I am less motivated to activities with physical exertion |  |  |  |  | |
| 4.4 Due to fatigue, I have difficulties continuing physical activities |  |  |  |  | |
| 4.5 Due to fatigue, I have difficulties making decisions |  |  |  |  | |
| 4.6 Due to fatigue, I have difficulties finishing tasks |  |  |  |  | |
| 4.7 Due to fatigue, I feel that my thinking is slower |  |  |  |  | |
| 4.8 Due to fatigue, I must reduce my physical activities |  |  |  |  | |

5.1 Were you admitted to the hospital when you had COVID-19 in January 2022?

Yes No

6.1 Have you had any infections since you had COVID-19/since January 2022?

Yes No

If Yes:

Once 2-3 times 4 times or more

**S4 Table** Characteristics of men and women in groups of cases (long COVID) and controls (never-infected)

|  | **Men** | | **Women** | |
| --- | --- | --- | --- | --- |
| **Characteristics** | Cases (n=43) | Controls (n=38) | Cases (n=134) | Controls (n=27) |
| Age, years, mean (SD) | 47.4 (13.1) | 58.4 (12.9) | 43.7 (13.9) | 63.7 (10.0) |
| Chronic disease, yes (%) | 19/43 (44.2) | 20/38 (52.6) | 69/134 (51.5) | 21/27 (77.8) |
| Daily medication use, yes (%) | 9/42 (21.4) | 20/37 (54.1) | 62/132 (47.0) | 19/27 (70.4) |
| Ever smoking, yes (%) | 28/42 (66.7) | 23/38 (60.1) | 68/133 (51.1) | 19/27 (70.4) |
| COVID vaccination,  2 or 3 (%) | 38/43 (88.4) | 38/38 (100) | 128/134 (95.5) | 27/27 (100) |
| Body mass index (kg/m^2^),  mean (SD) | 29.9 (4.6) | 30.0 (5.7) | 29.3 (6.3) | 29.2 (4.3) |

Abbreviations: SD: Standard Deviation

**S5 Table** Associations between individual persisting symptoms from the 24-item questionnaire and long COVID

|  | Odds ratio | 95% CI | P-value^*^ |
| --- | --- | --- | --- |
| Fatigue | 4.88 | 2.95-8.09 | <0.001 |
| Chills | 2.27 | 1.33-3.89 | <0.01 |
| Fever | 3.19 | 0.51-20.07 | 0.216 |
| Chest tightness | 5.55 | 2.25-13.72 | <0.001 |
| Sore throat | 8.81 | 2.56-30.31 | <0.001 |
| Shortness of breath | 4.79 | 2.43-9.45 | <0.001 |
| Dry cough | 2.17 | 1.27-3.72 | <0.01 |
| Nasal congestion | 2.06 | 1.29-3.27 | <0.01 |
| Sneezing | 1.85 | 1.14-3.00 | <0.05 |
| Wet cough | 1.49 | 0.97-2.31 | 0.070 |
| Rhinitis | 1.18 | 0.79-1.78 | 0.420 |
| Myalgia | 2.38 | 1.46-3.88 | <0.001 |
| Arthralgia | 1.74 | 1.17-2.61 | <0.001 |
| Concentration difficulties | 4.65 | 2.39-9.05 | <0.001 |
| Headache | 3.83 | 2.18-6.73 | <0.001 |
| Dizziness | 3.58 | 1.80-7.12 | <0.001 |
| Memory difficulties | 3.02 | 1.78-5.11 | <0.001 |
| Sleep difficulties | 2.20 | 1.43-3.38 | <0.001 |
| Affected taste | 3.44 | 1.53-7.73 | <0.01 |
| Affected smell | 2.43 | 1.38-4.27 | <0.01 |
| Anorexia | 1.44 | 0.65-3.18 | 0.370 |
| Nausea | 2.31 | 0.93-5.78 | 0.072 |
| Diarrhea | 1.62 | 0.93-2.84 | 0.090 |
| Rash | 0.89 | 0.46-1.73 | 0.737 |

Abbreviations: CI: Confidence Interval

^*^Multivariable logistic regression analysis adjusted for sex, age, daily medication, and chronic disease.

**S6 Table** White blood cell counts in men (LC group and NI group)

|  | **Men** | | |  |
| --- | --- | --- | --- | --- |
|  | Group | Mean* | Range* | SD |
| Leukocytes | LC | 6.8 | 3.1-13.2 | 2.0 |
|  | NI | 7.0 | 4.6-11.0 | 1.6 |
| Neutrophils | LC | 3.7 | 1.4-8.4 | 1.4 |
|  | NI | 3.9 | 2.3-7.0 | 1.3 |
| Basophils | LC | 0.05 | 0.00-0.1 | 0.5 |
|  | NI | 0.05 | 0.00-0.1 | 0.5 |
| Eosinophils | LC | 0.2 | 0.00-0.8 | 0.1 |
|  | NI | 0.2 | 0.00-0.5 | 0.1 |
| Lymphocytes | LC | 2.3 | 1.0-6.0 | 0.9 |
|  | NI | 2.2 | 1.0-3.7 | 0.6 |
| Monocytes | LC | 0.6 | 0.3-1.3 | 0.2 |
|  | NI | 0.6 | 0.4-1.1 | 0.2 |

Abbreviations: LC: Long COVID; NI: Never infected; SD: Standard Deviation

*Cell counts are 10^9^ per Liter.

**S7 Table** White blood cell counts in women (LC group and NI group)

|  | **Women** |  |  |  |
| --- | --- | --- | --- | --- |
|  | Group | Mean* | Range* | SD |
| Leukocytes | LC | 6.7 | 3.0-12.6 | 1.8 |
|  | NI | 7.2 | 3.5-11.2 | 2.3 |
| Neutrophils | LC | 3.8 | 1.0-9.1 | 1.4 |
|  | NI | 4.1 | 1.7-6.7 | 1.7 |
| Basophils | LC | 0.04 | 0.00-0.1 | 0.05 |
|  | NI | 0.06 | 0.00-0.1 | 0.05 |
| Eosinophils | LC | 0.2 | 0.00-1.0 | 0.2 |
|  | NI | 0.2 | 0.00-0.8 | 0.2 |
| Lymphocytes | LC | 2.1 | 0.9-3.6 | 0.6 |
|  | NI | 2.3 | 0.9-3.9 | 0.8 |
| Monocytes | LC | 0.5 | 0.2-1.4 | 0.2 |
|  | NI | 0.6 | 0.3-1.4 | 0.2 |

Abbreviations: LC: Long COVID; NI: Never infected; SD: Standard Deviation

*Cell counts are 10^9^ per Liter.

**S1 Fig.** The distribution of the self-reported questions in the mental health questionnaire in the long-COVID group (first panel) and the never-infected group (second panel)

**
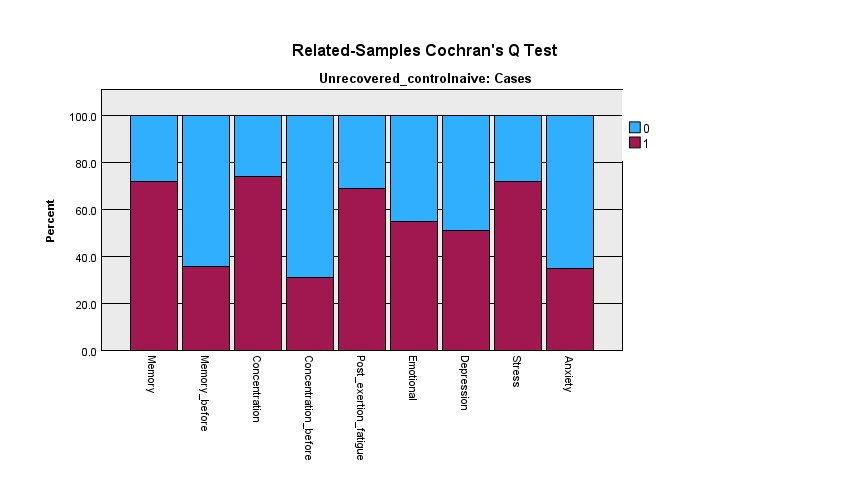
**0=no, 1=yes; Related-samples Cochran’s Q test: p<0.001

**
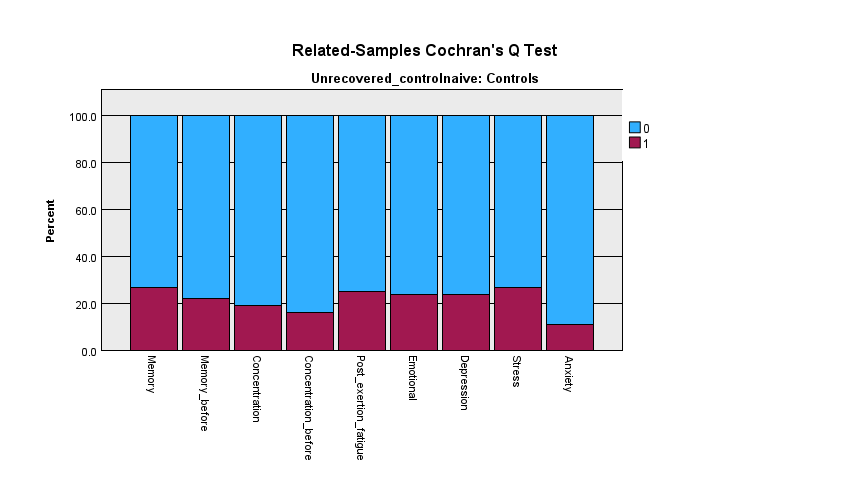
**

0=no, 1=yes; Related-samples Cochran’s Q test: p=0.09

**S2 Fig.** The distribution of the self-reported questions in the fatigue impact questionnaire in the long-COVID group (first panel) and the never-infected group (second panel)

**
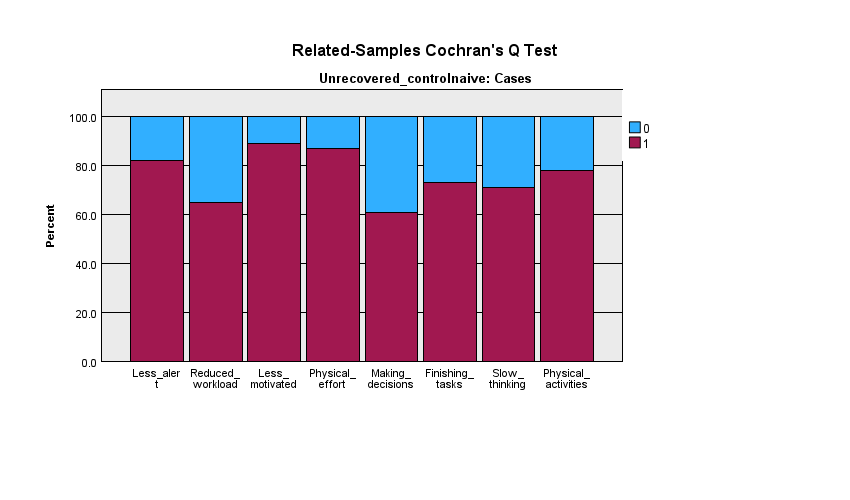
**

0=no, 1=yes; Related-samples Cochran’s Q test: p<0.001

**
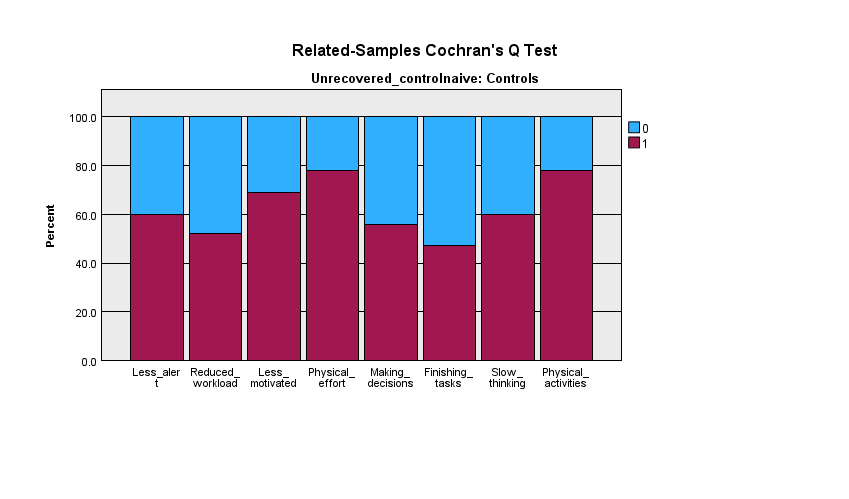
**0=no, 1=yes; Related-samples Cochran’s Q test: p=0.1
