# Supplementary material for: Persistent symptoms, cognitive impairment, and clinical predictors of long COVID one year after Omicron infection: A clinical case–control study from the Faroe Islands
Source: PLoS One. 2026 Jun 16;21(6):e0351564. doi: 10.1371/journal.pone.0351564 (PMC13271491; doi:10.1371/journal.pone.0351564)
Supplement: Attachment — Submitted filename: Response to reviewers.docx [file pone.0351564.s002.docx]

**Response to reviewers**	Tórshavn, 18 May 2026

Thank you for your comments on the submitted manuscript. We have revised the manuscript in response to the recommended comments and have also added more detailed information from the results. We hope that this has improved the manuscript.

Reviewer #1:

1. Statistical power of comparisons: Unfortunately, the number of controls was low. We aimed for an equal number in both groups (200+200). Still, most of the controls from the original group reported having already been infected, and we also found that quite a few had antibodies indicative of prior infection. We have added a comment on this in the Discussion, line numbers 395-399.

2. Selection process: The Methods section has been revised so that it includes subheadings explaining the different steps in the study. The text is hopefully clearer now. Line numbers 87-102.

3. Clinical relevance of the laboratory findings: We do not know why there was a difference in WBC in women. The result could be due to residual confounding (e.g chronic disease) as suggested in the Discussion, line numbers 375-378.

Reviewer #2:

1. Case-control ratio: Unfortunately, the number of controls was low. We aimed for an equal number in both groups. Still, most of the controls from the original group reported having already been infected, and we also found that quite a few had antibodies indicative of prior infection. We have added a comment on this in the Discussion, line numbers 390-395. Including the seropositive controls was an option, as in previous studies, but we chose to use an immunoassay on our samples, believing it would improve the accuracy of the statistical analysis.

2. Power calculations: We aimed for a number of 200 individuals in each group to achieve a power of 80% chance of detecting a small-to-moderate effect. A comment on this has been added to the section on statistical analysis, line numbers 170-171.

3. Confounders: We have found an error in our description of the confounder selection. The selection was not based on univariable analysis; on the other hand, a multivariable logistic regression was used using backward selection. This has now been corrected, line numbers 160-161.

4. Multiple comparisons: It is correct that we have not considered multiple comparisons in our analysis and discussion. Regarding the questionnaires, we have mostly focused on the total scores and not so much on the scores from the individual questions when comparing the groups. On the other hand, when discussing the results from the blood samples, we could have done this, but chose not to, as the analysis was exploratory, as stated in the statistical section, line number 176. We hope that this is a satisfactory explanation.

5. WBC counts: Yes, this is true, and maybe these results should have been omitted. We cannot really explain this difference, but it is probably due to residual confounding, possibly through chronic disease/medication, increasing WBC counts in the controls. We have added some additional comments on this (line numbers 375-378).

6. The reference has been corrected.

Yours sincerely,

Gunnhild Helmsdal, MD

Department of Research, National Hospital, Sigmundargøta 5, FO-100 Tórshavn, Faroe Islands

Telephone: +298 263010

E-mail: gunhe@ls.fo
