# Supplementary material for: Persistent symptoms, cognitive impairment, and clinical predictors of long COVID one year after Omicron infection: A clinical case–control study from the Faroe Islands
Source: PLoS One. 2026 Jun 16;21(6):e0351564. doi: 10.1371/journal.pone.0351564 (PMC13271491; doi:10.1371/journal.pone.0351564)
Supplement: Peer review history

# Peer review: Decision Letter 0

**DOI:** 10.1371/journal.pone.0351564.r001

**Authors:** Petros Isaakidis

7 Apr 2026

PONE-D-25-65362

Persistent Symptoms, Cognitive Impairment, and Clinical Predictors of Long COVID One Year After Omicron Infection: A Clinical Case–Control Study from the Faroe Islands

PLOS One

Dear Dr. Helmsdal,

Thank you for submitting your manuscript to PLOS ONE. After careful consideration, we feel that it has merit but does not fully meet PLOS ONE’s publication criteria as it currently stands. Therefore, we invite you to submit a revised version of the manuscript that addresses the points raised during the review process.

The manuscript might benefit from a clearer justification and discussion of the statistical methods, particularly the case–control ratio, the model building approach in detail, as well as the handling of confounders. These should be explicitly detailed in both the Methods and Discussion sections. Providing further context for power limitations, multiple comparisons, and the interpretation of statistically significant but clinically modest differences for example would strengthen the  overall rigor of the study.

Please submit your revised manuscript by May 22 2026 11:59PM. If you will need more time than this to complete your revisions, please reply to this message or contact the journal office at plosone@plos.org. When you're ready to submit your revision, log on to https://www.editorialmanager.com/pone/ and select the 'Submissions Needing Revision' folder to locate your manuscript file.

Please include the following items when submitting your revised manuscript:

- A letter that responds to each point raised by the academic editor and reviewer(s). You should upload this letter as a separate file labeled 'Response to Reviewers'.
- A marked-up copy of your manuscript that highlights changes made to the original version. You should upload this as a separate file labeled 'Revised Manuscript with Track Changes'.
- An unmarked version of your revised paper without tracked changes. You should upload this as a separate file labeled 'Manuscript'.

If you would like to make changes to your financial disclosure, please include your updated statement in your cover letter. Guidelines for resubmitting your figure files are available below the reviewer comments at the end of this letter.

If applicable, we recommend that you deposit your laboratory protocols in protocols.io to enhance the reproducibility of your results. Protocols.io assigns your protocol its own identifier (DOI) so that it can be cited independently in the future. For instructions see: https://journals.plos.org/plosone/s/submission-guidelines#loc-laboratory-protocols. Additionally, PLOS ONE offers an option for publishing peer-reviewed Lab Protocol articles, which describe protocols hosted on protocols.io. Read more information on sharing protocols at https://plos.org/protocols?utm_medium=editorial-email&utm_source=authorletters&utm_campaign=protocols.

As the corresponding author, your ORCID iD is verified in the submission system and will appear in the published article. PLOS supports the use of ORCID, and we encourage all coauthors to register for an ORCID iD and use it as well. Please encourage your coauthors to verify their ORCID iD within the submission system before final acceptance, as unverified ORCID iDs will not appear in the published article. *Only*   the individual author can complete the verification step; PLOS staff *cannot*   verify ORCID iDs on behalf of authors.

We look forward to receiving your revised manuscript.

Kind regards,

Petros Isaakidis

Academic Editor

PLOS One

**Journal Requirements:**

When submitting your revision, we need you to address these additional requirements.

1. Please ensure that your manuscript meets PLOS ONE's style requirements, including those for file naming. The PLOS ONE style templates can be found at

https://journals.plos.org/plosone/s/file?id=wjVg/PLOSOne_formatting_sample_main_body.pdf and

https://journals.plos.org/plosone/s/file?id=ba62/PLOSOne_formatting_sample_title_authors_affiliations.pdf

2. In the online submission form you indicate that your data is not available for proprietary reasons and have provided a contact point for accessing this data. Please note that your current contact point is a co-author on this manuscript. According to our Data Policy, the contact point must not be an author on the manuscript and must be an institutional contact, ideally not an individual. Please revise your data statement to a non-author institutional point of contact, such as a data access or ethics committee, and send this to us via return email. Please also include contact information for the third party organization, and please include the full citation of where the data can be found.

3. Please upload a new copy of Figures 1A and 1B as the detail is not clear. Please follow the link for more information:  https://journals.plos.org/plosone/s/figures

4. If the reviewer comments include a recommendation to cite specific previously published works, please review and evaluate these publications to determine whether they are relevant and should be cited. There is no requirement to cite these works unless the editor has indicated otherwise.

[Note: HTML markup is below. Please do not edit.]

Reviewers' comments:

Reviewer's Responses to Questions

**Comments to the Author**

1. Is the manuscript technically sound, and do the data support the conclusions?

The manuscript must describe a technically sound piece of scientific research with data that supports the conclusions. Experiments must have been conducted rigorously, with appropriate controls, replication, and sample sizes. The conclusions must be drawn appropriately based on the data presented.

Reviewer #1: Yes

Reviewer #2: Yes

**********

2. Has the statistical analysis been performed appropriately and rigorously?

Reviewer #1: Yes

Reviewer #2: I Don't Know

**********

3. Have the authors made all data underlying the findings in their manuscript fully available?

The PLOS Data policy requires authors to make all data underlying the findings described in their manuscript fully available without restriction, with rare exception (please refer to the Data Availability Statement in the manuscript PDF file). The data should be provided as part of the manuscript or its supporting information, or deposited to a public repository. For example, in addition to summary statistics, the data points behind means, medians and variance measures should be available. If there are restrictions on publicly sharing data—e.g. participant privacy or use of data from a third party—those must be specified.

Reviewer #1: Yes

Reviewer #2: No

**********

4. Is the manuscript presented in an intelligible fashion and written in standard English?

PLOS ONE does not copyedit accepted manuscripts, so the language in submitted articles must be clear, correct, and unambiguous. Any typographical or grammatical errors should be corrected at revision, so please note any specific errors here.

Reviewer #1: Yes

Reviewer #2: Yes

**********

5. Review Comments to the Author

Please use the space provided to explain your answers to the questions above. You may also include additional comments for the author, including concerns about dual publication, research ethics, or publication ethics. (Please upload your review as an attachment if it exceeds 20,000 characters)

Reviewer #1: Overall, this manuscript addresses an important topic and provides useful information about persistent symptoms after Omicron infection. The study design is clear and includes a control group of individuals who were not previously infected, which strengthens the analysis. The authors used questionnaires, clinical examination, and laboratory tests, providing a comprehensive evaluation of participants.

The results are presented clearly and the statistical methods appear appropriate. The findings regarding fatigue, cognitive symptoms, and mental impairment are consistent with previous studies on long COVID and add additional data from a well-defined population.

However, a few points could be clarified. The sample size of the control group is relatively small, which may limit the statistical power of the comparisons. It would also be helpful if the authors further explain the selection process for participants included in the clinical follow-up to better understand potential selection bias. In addition, more discussion on the clinical relevance of the laboratory findings, particularly the differences in white blood cell counts, would strengthen the interpretation of the results.

The manuscript is well organized and contributes meaningful data to the understanding of long COVID after Omicron infection.

Reviewer #2: Overall, this is a well thought out and well-written paper investigating risk factors for long COVID after the Omicron wave in a relatively isolated population in the Faroe islands. This paper describes risk factors in a society with access to modern health care and a relatively high national income. Several factors were identified that were associated with long COVID in this analysis. However, the discussion section particularly on limitations of the study are glossed over even with more uncommon methodologies.

Language:

Generally written appropriately throughout. Several instances of "Covid-19" vs. "COVID-19" in other places -- would keep this consistent throughout.

Intro:

No issues

Findings/discussion:

Overall describes the detais of case: control selection adequately, as well as the utilization of the survey and clinical data.

- The utilization of essentially 3 cases: 1 control is methodologically uncommon in the literature (usually controls up to 4 per case are general practice as increasing power/ability to detect a difference). Aside from the practical limitations related to the low seronegative rate, this is not addressed in either the methods section or in the discussion where it warrants further comment. Furthermore, the controls as being a highly selected group was not explored as a limitation in the contextualization of the findings.

- As such, there is no comment or discussion on power calculation or sample size.

- The use of confounds with p<0.05 in a univariate model for their multivariate model is potentially problematic and exposes to potential biases without great explanation why this approach was used. Usually confounders are determined by the expertise available. This reviewer will fully acknowledge a lack of expertise in statistics, but I am familiar with recommendations from scientific societies regarding methodological approaches in this setting. This is essentially a step-wise approach to confounding.

- It does not appear the authors have considered multiple comparisons across the many different variables and hypothesis tests between symptoms, neuropsychiatric symptoms

- The authors spent a decent portion of the results and discussion on WBC count differences between LC and NI groups, particularly related to differencs between sex. Overall, the differences as outlined demonstrate statistical significance but all well within a normal leukocyte count overall, yes the medians are different, but still well within a normal WBC count and this is not addressed in the discussion --- some readers might be asking whether or not these even matters. Why were the differences in WBC and sex not explored as an interaction term?

- Similarly, the focus on basophil significance without an explanation of a hypothesis -- it seems odd to this reviewer that basophils would be more significant than eosinophils. Again, this is not addressed in the discussion -- to me for these findings are statistically significant but there might not be a broader significance since no hypothesis or future direction is discussed.

- Lines 294-298 -- check reference, it sounds like it's Iba et al, but the refence of 28 is aksura --- I suspect this should be 29.

**********

6. PLOS authors have the option to publish the peer review history of their article (what does this mean?). If published, this will include your full peer review and any attached files.

If you choose “no”, your identity will remain anonymous but your review may still be made public.

**Do you want your identity to be public for this peer review?**   For information about this choice, including consent withdrawal, please see our Privacy Policy.

Reviewer #1: **Yes:**   Yuriy Bisyuk

Reviewer #2: No

**********

[NOTE: If reviewer comments were submitted as an attachment file, they will be attached to this email and accessible via the submission site. Please log into your account, locate the manuscript record, and check for the action link "View Attachments". If this link does not appear, there are no attachment files.]

To ensure your figures meet our technical requirements, please review our figure guidelines: https://journals.plos.org/plosone/s/figures

You may also use PLOS’s free figure tool, NAAS, to help you prepare publication quality figures: https://journals.plos.org/plosone/s/figures#loc-tools-for-figure-preparation.

NAAS will assess whether your figures meet our technical requirements by comparing each figure against our figure specifications.

# Author comment: Author response to Decision Letter 1

**DOI:** 10.1371/journal.pone.0351564.r002

19 May 2026

Response to reviewers Tórshavn, 18 May 2026

Thank you for your comments on the submitted manuscript. We have revised the manuscript in response to the recommended comments and have also added more detailed information from the results. We hope that this has improved the manuscript.

Reviewer #1:

1. Statistical power of comparisons: Unfortunately, the number of controls was low. We aimed for an equal number in both groups (200+200). Still, most of the controls from the original group reported having already been infected, and we also found that quite a few had antibodies indicative of prior infection. We have added a comment on this in the Discussion, line numbers 395-399.

2. Selection process: The Methods section has been revised so that it includes subheadings explaining the different steps in the study. The text is hopefully clearer now. Line numbers 87-102.

3. Clinical relevance of the laboratory findings: We do not know why there was a difference in WBC in women. The result could be due to residual confounding (e.g chronic disease) as suggested in the Discussion, line numbers 375-378.

Reviewer #2:

1. Case-control ratio: Unfortunately, the number of controls was low. We aimed for an equal number in both groups. Still, most of the controls from the original group reported having already been infected, and we also found that quite a few had antibodies indicative of prior infection. We have added a comment on this in the Discussion, line numbers 390-395. Including the seropositive controls was an option, as in previous studies, but we chose to use an immunoassay on our samples, believing it would improve the accuracy of the statistical analysis.

2. Power calculations: We aimed for a number of 200 individuals in each group to achieve a power of 80% chance of detecting a small-to-moderate effect. A comment on this has been added to the section on statistical analysis, line numbers 170-171.

3. Confounders: We have found an error in our description of the confounder selection. The selection was not based on univariable analysis; on the other hand, a multivariable logistic regression was used using backward selection. This has now been corrected, line numbers 160-161.

4. Multiple comparisons: It is correct that we have not considered multiple comparisons in our analysis and discussion. Regarding the questionnaires, we have mostly focused on the total scores and not so much on the scores from the individual questions when comparing the groups. On the other hand, when discussing the results from the blood samples, we could have done this, but chose not to, as the analysis was exploratory, as stated in the statistical section, line number 176. We hope that this is a satisfactory explanation.

5. WBC counts: Yes, this is true, and maybe these results should have been omitted. We cannot really explain this difference, but it is probably due to residual confounding, possibly through chronic disease/medication, increasing WBC counts in the controls. We have added some additional comments on this (line numbers 375-378).

6. The reference has been corrected.

Yours sincerely,

Gunnhild Helmsdal, MD

Department of Research, National Hospital, Sigmundargøta 5, FO-100 Tórshavn, Faroe Islands

Telephone: +298 263010

E-mail: gunhe@ls.fo

Attachment Submitted filename: Response to reviewers.docx

# Editor report: Decision Letter 1

**DOI:** 10.1371/journal.pone.0351564.r003

**Authors:** Petros Isaakidis

28 May 2026

Persistent symptoms, cognitive impairment, and clinical predictors of long COVID one year after Omicron infection: A clinical case–control study from the Faroe Islands

PONE-D-25-65362R1

Dear Dr. Helmsdal,

We’re pleased to inform you that your manuscript has been judged scientifically suitable for publication and will be formally accepted for publication once it meets all outstanding technical requirements.

Within one week, you’ll receive an e-mail detailing the required amendments. When these have been addressed, you’ll receive a formal acceptance letter and your manuscript will be scheduled for publication.

An invoice will be generated when your article is formally accepted. Please note, if your institution has a publishing partnership with PLOS and your article meets the relevant criteria, all or part of your publication costs will be covered. Please make sure your user information is up-to-date by logging into Editorial Manager at Editorial Manager® and clicking the ‘Update My Information' link at the top of the page. For questions related to billing, please contact billing support.

If your institution or institutions have a press office, please notify them about your upcoming paper to help maximize its impact. If they’ll be preparing press materials, please inform our press team as soon as possible -- no later than 48 hours after receiving the formal acceptance. Your manuscript will remain under strict press embargo until 2 pm Eastern Time on the date of publication. For more information, please contact onepress@plos.org.

Kind regards,

Petros Isaakidis

Academic Editor

PLOS One

Additional Editor Comments (optional):

Reviewers' comments:

# Editor report: Acceptance letter

**DOI:** 10.1371/journal.pone.0351564.r004

**Authors:** Petros Isaakidis

PONE-D-25-65362R1

PLOS One

Dear Dr. Helmsdal,

I'm pleased to inform you that your manuscript has been deemed suitable for publication in PLOS One. Congratulations! Your manuscript is now being handed over to our production team.

At this stage, our production department will prepare your paper for publication. This includes ensuring the following:

* All references, tables, and figures are properly cited

* All relevant supporting information is included in the manuscript submission,

* There are no issues that prevent the paper from being properly typeset

You will receive further instructions from the production team, including instructions on how to review your proof when it is ready. Please keep in mind that we are working through a large volume of accepted articles, so please give us a few days to review your paper and let you know the next and final steps.

Lastly, if your institution or institutions have a press office, please let them know about your upcoming paper now to help maximize its impact. If they'll be preparing press materials, please inform our press team within the next 48 hours. Your manuscript will remain under strict press embargo until 2 pm Eastern Time on the date of publication. For more information, please contact onepress@plos.org.

You will receive an invoice from PLOS for your publication fee after your manuscript has reached the completed accept phase. If you receive an email requesting payment before acceptance or for any other service, this may be a phishing scheme. Learn how to identify phishing emails and protect your accounts at https://explore.plos.org/phishing.

If we can help with anything else, please email us at customercare@plos.org.

Thank you for submitting your work to PLOS ONE and supporting open access.

Kind regards,

PLOS ONE Editorial Office Staff

on behalf of

Dr. Petros Isaakidis

Academic Editor

PLOS One
